# ATRX represses alternative lengthening of telomeres

**DOI:** 10.18632/oncotarget.3846

**Published:** 2015-04-15

**Authors:** Christine E. Napier, Lily I. Huschtscha, Adam Harvey, Kylie Bower, Jane R. Noble, Eric A. Hendrickson, Roger R. Reddel

**Affiliations:** ^1^ Cancer Research Unit, Children's Medical Research Institute, The University of Sydney, Westmead, NSW, Australia; ^2^ Department of Biochemistry, Molecular Biology and Biophysics, University of Minnesota Medical School, Minneapolis, MN, USA

**Keywords:** ATRX, ALT, telomere, immortalization

## Abstract

The unlimited proliferation of cancer cells requires a mechanism to prevent telomere shortening. Alternative Lengthening of Telomeres (ALT) is an homologous recombination-mediated mechanism of telomere elongation used in tumors, including osteosarcomas, soft tissue sarcoma subtypes, and glial brain tumors. Mutations in the ATRX/DAXX chromatin remodeling complex have been reported in tumors and cell lines that use the ALT mechanism, suggesting that ATRX may be an ALT repressor. We show here that knockout or knockdown of ATRX in mortal cells or immortal telomerase-positive cells is insufficient to activate ALT. Notably, however, in SV40-transformed mortal fibroblasts ATRX loss results in either a significant increase in the proportion of cell lines activating ALT (instead of telomerase) or in a significant decrease in the time prior to ALT activation. These data indicate that loss of ATRX function cooperates with one or more as-yet unidentified genetic or epigenetic alterations to activate ALT. Moreover, transient ATRX expression in ALT-positive/ATRX-negative cells represses ALT activity. These data provide the first direct, functional evidence that ATRX represses ALT.

## INTRODUCTION

Telomeres are repetitive DNA structures located at the ends of chromosomes that shorten with each cell division [1]. When telomeres become sufficiently shortened, the cell will enter a permanent proliferation arrest termed senescence. In order to become immortalized, cancer cells require a mechanism of telomere length maintenance. There are two mechanisms currently known to maintain telomere length in human cells: the reverse transcriptase enzyme telomerase and the recombination-mediated DNA synthesis mechanism called Alternative Lengthening of Telomeres (ALT) [2, 3]. Telomerase activation can occur through numerous means, including loss of repressors, transcriptional upregulation or amplification of the genes encoding a telomerase subunit, TERT and/or TERC, and mutations in the TERT promoter (reviewed in [4]). Somatic cell hybridization analyses showed that ALT activation occurs through loss of one or more repressor molecules that are present in normal somatic cells and in telomerase positive cells [3, 5]. More recently, it was found that inactivating mutations in one or other of the genes encoding the ATRX/DAXX chromatin remodeling complex, most commonly ATRX, are very common in ALT-positive tumors and cell lines [6-10]. ATRX loss was also highly correlated with ALT in a panel of 19 ALT/telomerase cell line hybrids [11]. ATRX has been proposed to have numerous diverse functions, including chromatin remodeling, viral resistance, and fidelity of chromatin separation during cell division, as well as binding to tandem DNA repeats [12-15]. Nonetheless, these data suggest the hypothesis that ATRX may be an ALT repressor.

In human cell culture models of immortalization, the first events required for this process usually involve inactivation of the p53 and pRB/p16INK4a tumor suppressor pathways by, for example, expression of one or more oncoproteins such as the simian virus 40 (SV40) large T antigen, which allows the cells to continue dividing beyond the point at which control cultures become senescent. After a finite number of additional cell divisions, however, the cells cease proliferation and the culture enters crisis, until a rare cell (approximately 1 in 10^7^ fibroblasts and 1 in 10^5^ epithelial cells) escapes crisis through spontaneous activation of a telomere lengthening mechanism (TLM), and the cell culture becomes immortal [16-19]. In fibroblast cultures, the probability that ALT will be activated is approximately the same as for activation of telomerase, whereas epithelial cells are much more likely to activate telomerase; this reflects the situation in human cancers where carcinomas are usually telomerase-positive and ALT is most prevalent in cancers of mesenchymal origin [20]. It has long been assumed that activation of a TLM and escape from crisis involves spontaneous genetic or epigenetic events, that different events are required for activation of ALT and telomerase and, based on the frequency of their occurrence, that activation of each TLM requires one or more “hits”.

In this study, we determined whether loss of ATRX is one of the genetic events involved in activating ALT, comparing epithelial cells with fibroblasts. To this end, we genetically disrupted ATRX in immortal telomerase positive epithelial cells to determine whether loss of ATRX was sufficient to activate ALT. As these cells did not activate the ALT mechanism, we went on to knock down ATRX in SV40-transformed pre-crisis cells. In cells of epithelial origin, a reduction in ATRX also did not lead to ALT activation. However, in pre-crisis SV40-transformed fibroblasts derived from two different sources, the knockdown of ATRX led either to an increased frequency of ALT activation or a decrease in the time of crisis prior to immortalization via the ALT mechanism. Just as importantly, the transient expression of exogenous ATRX in three independent ALT-positive, ATRX-negative cell lines led to a reduction in C-circles and ALT-associated PML bodies (APBs), two markers of ALT activity. These data provide the first functional evidence that ATRX acts as a repressor of the ALT mechanism in cells of mesenchymal origin.

## RESULTS

### ATRX gene knockout does not activate ALT in a telomerase-positive cell line

**Figure 1:**
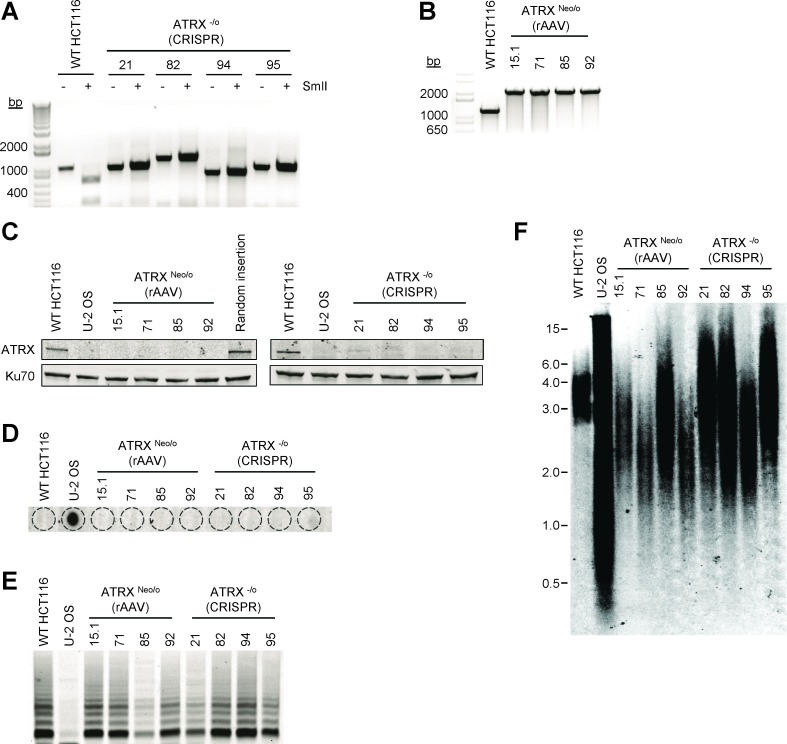
An ATRX knockout is compatible with telomerase activity **A.** PCR across the region of the ATRX gene targeted by CRISPR/Cas9 treatment in HCT116 cells shows that all clones contain modifications that result in disruption of the *Sml*I restriction enzyme recognition sequence compared to wild-type (WT). Note that ATRX is located on the X chromosome and HCT116 is derived from a male, making the gene hemizygous. **B.** PCR confirming correct gene targeting by rAAV using ATRX IntF and ATRX IntR primers (Supplementary Table 1). Wild-type cells should show a band of 1217 bp, while correctly targeted clones yield of band of 2186 bp. **C.** Western blot analysis was conducted to confirm the lack of ATRX expression in all CRISPR/Cas9 and rAAV correctly-targeted HCT116 clones. Ku70 was used as a loading control. Telomere maintenance status was analyzed (**D.** C-circle, **E.** TRAP and **F.** TRF assays) in both wild-type and ATRX-knockout HCT116 cells. DNA from U-2 OS cells was used as a positive control for the C-circle and TRF assays and a negative control for the TRAP assay. All HCT116 clones were telomerase-positive/ALT-negative by these criteria.

Previous findings in tumor cells and cell lines have shown a correlation between ALT and absence of ATRX at the protein or gene level. To determine whether knockout of ATRX in the telomerase-positive epithelial cell line HCT116 activated the ALT mechanism, we used two targeting strategies: CRISPR (clustered, regularly interspaced, short palindromic repeats)/CRISPR-associated systems (Cas)9 and recombinant adeno-associated virus (rAAV). Following single cell cloning of HCT116 cells co-transfected with CRISPR/Cas9 [21], correctly targeted clones were identified by restriction enzyme analysis of a PCR product that encompassed the CRISPR target (Figure 1A). HCT116 clones with a disrupted ATRX gene were resistant to digestion with the *Sml*I restriction enzyme due to removal of the *Sml*I recognition site, which was verified by Sanger sequencing. As a second method to knock out ATRX, an rAAV gene targeting vector was constructed to replace exon 5 of ATRX, and four clones resistant to G418 were analyzed for correct integration of the construct using primers that flank the proposed integration site (Figure 1B). Absence of ATRX protein expression in knockout clones constructed using either the CRISPR/Cas9 or rAAV method was confirmed by Western blot (Figure 1C).

We analyzed the ATRX knockout HCT116 cells to determine whether the ALT mechanism was activated. Similar to wild-type HCT116 cells, ATRX knockout HCT116 cells were negative for C-circles, partially single-stranded circles of C-rich telomeric DNA that are highly associated with the ALT mechanism [22] (Figure 1D). We also found all ATRX knockout HCT116 clones expressed telomerase activity as determined by the TRAP assay (Figure 1E). Telomere length was assessed by Southern blot 29 population doublings (PDs) after cloning and did not show the elongated and heterogeneous telomere length profile that is characteristic of cells that utilize ALT (Figure 1F). These data demonstrate that the knockout of ATRX in telomerase-positive carcinoma cells is not sufficient to activate the ALT mechanism.

### ATRX depletion in epithelial cells does not promote ALT activation

**Figure 2:**
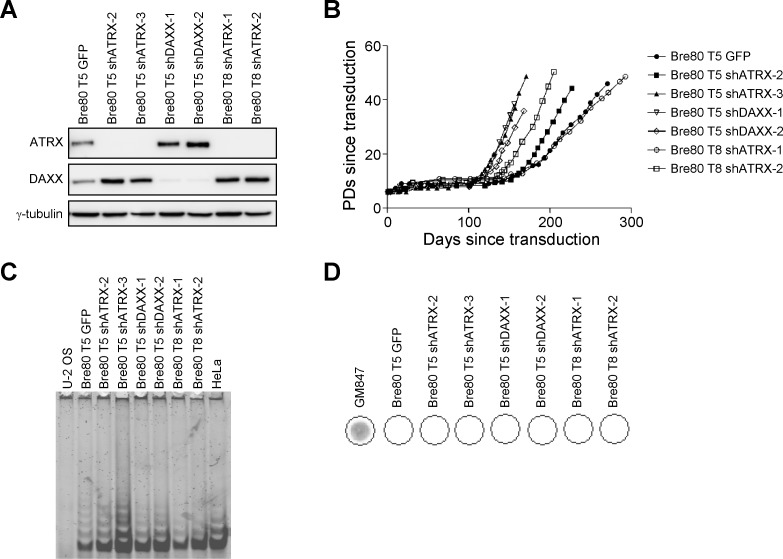
Depletion of ATRX does not induce ALT in epithelial cells **A.** the indicated Bre80 epithelial cultures were analyzed for ATRX and DAXX protein expression at a time point after immortalization; γ-tubulin was used as a loading control. **B.** growth curves of each immortalized Bre80 T5 and Bre80 T8 cell line. The number of days in culture since transduction is indicated on the x-axis, while the y-axis indicates the number of PDs the culture has undergone post-transduction. **C.** telomerase activity was assessed in each immortal culture using the TRAP assay. U-2 OS and HeLa cell lysates were used as negative and positive controls, respectively. **D.** the presence of C-circles in each immortal Bre80 T5 and Bre80 T8 cell line was determined. DNA from GM847 cells was used as a positive control.

Epithelial-derived cell lines and tumors preferentially activate telomerase [20, 23], and cells that are telomerase-positive generally express ATRX [7, 11]. We therefore determined whether loss of ATRX promoted ALT activation in SV40-transformed mammary epithelial cells. Two epithelial cell strains derived from individual SV40-transformation events, Bre80 T5 and Bre80 T8, were transduced with an empty vector (vector), scrambled control shRNA (sc), green fluorescent protein (GFP), or an shRNA targeting ATRX or DAXX. One of five control cultures (Bre80 T5 GFP; Table 1), and six of ten shATRX or shDAXX cultures emerged from crisis. Each immortal line was assessed for ATRX and DAXX expression (Figure 2A). Bre80 T5 GFP cells expressed ATRX and DAXX protein. The four shATRX and two shDAXX immortal lines lacked ATRX and DAXX expression, respectively. All immortal cultures exhibited a period of crisis that ranged from 64 to 166 days (Figure 2B). All immortal cultures were positive by the TRAP assay for telomerase activity (Figure 2C) and negative for the presence of C-circles (Figure 2D), together indicating that all seven cultures activated telomerase. These results demonstrate that ATRX depletion in epithelial cells does not promote ALT activation. Furthermore, these data, together with the HCT116 ATRX knockout data, indicate that neither telomerase activity nor activation of telomerase is dependent upon the presence of ATRX.

**Table 1:** Pre- and post-crisis protein and telomere lengthening mechanism characterization of cell strains and cell lines

		Pre-crisis			Post-crisis					
		Protein^a^						Protein^a^		
Parental cell strain	Expression construct	ATRX	DAXX	Days in crisis^b^	C circles^c^	TRAP^d^	TRF^e^	ATRX	DAXX	TLM^f^
Bre80 T5	GFP	nd	nd	166	−	+	nd	+	+	TEL
	vector-1	nd	nd	136*						−
	vector-2	nd	nd	264*						−
	vector-3	nd	nd	264*						−
	sc	nd	nd	187*						−
	shATRX-1	nd	nd	109*						−
	shATRX-2	nd	nd	107	−	+	nd	−	+	TEL
	shATRX-3	nd	nd	64	−	+	nd	−	+	TEL
	shDAXX-1	nd	nd	98	−	+	nd	+	−	TEL
	shDAXX-2	nd	nd	66	−	+	nd	+	−	TEL
Bre80 T8	shATRX-1	nd	nd	92	−	+	−	−	+	TEL
	shATRX-2	nd	nd	97	−	+	−	−	+	TEL
	shATRX-3	nd	nd	102*						−
	shATRX-4	nd	nd	102*						−
	shATRX-5	nd	nd	102*						−
Fre80-3T	sc1	+	+	65	−	+	nd	+	+	TEL
	sc2	+	+	23	−	+	nd	+	+	TEL
	sc3	+	+	124*						−
	sc4	+	+	22	−	+	nd	+	+	TEL
	sc5	+	+	99	−	+	nd	+	+	TEL
	sc6	+	+	30	−	+	nd	+	+	TEL
	shATRX-1	nd	nd	62	+	−	nd	−	+	ALT
	shATRX-2	−	+	125*						−
	shATRX-3	−	+	99	−	+	nd	−	+	TEL
	shATRX-4	−	+	158*						−
	shATRX-5	−	+	125*						−
	shATRX-6	−	+	158*						−
	shATRX-7	−	+	0	−	+	nd	low	+	TEL
Fre80-4Tii	none (parental)	+	+	60	+	−	ALT	+	+	ALT
	sc1	nd	nd	87*						−
	sc2	+	+	87*						−
	sc3	+	+	87*						−
	sc4	+	+	78*						−
	sc5	+	+	82*						−
	sc6	+	+	82*						−
	shATRX-1	nd	nd	31	+	−	ALT	−	+	ALT
	shATRX-2	nd	nd	93	+	−	nd	−	nd	ALT
	shATRX-3	nd	nd	112	+	−	ALT	−	+	ALT
	shATRX-4	−	+	14	+	−	ALT	−	+	ALT
	shATRX-5	−	+	27	+	−	nd	−	+	ALT
	shATRX-6	−	+	44	+	−	nd	−	+	ALT
	shATRX-7	−	+	57	+	−	nd	−	+	ALT
	shATRX-8	−	+	46	+	−	nd	−	+	ALT
	shATRX-9	−	+	42	+	−	nd	−	+	ALT
JFCF-6/T.1/P	none (parental)	+	+	44	+	−	+	−	+	ALT
	vector	+	+	13	+	−	+	−	+	ALT
	sc1	+	+	42	+	−	+	+	+	ALT
	sc2	+	+	78	+	−	+	−	+	ALT
	shATRX-1	−	+	9	+	−	+	−	+	ALT
	shATRX-2	−	+	0	+	−	+	−	+	ALT
	shATRX-3	−	+	0	+	−	+	−	+	ALT
	shATRX-4	−	+	0	+	−	+	−	+	ALT
	shDAXX	+	−	0	+	−	+	+	−	ALT
JFCF-6/T.5K	none (parental)	+	+	0	+	−	+	−	+	ALT
	vector	+	+	48	+	−	+	−	+	ALT
	sc1	+	+	46	+	−	+	−	+	ALT
	sc2	+	+	0	+	−	+	−	+	ALT
	shATRX-1	−	+	12	+	−	+	−	+	ALT
	shATRX-2	−	+	26	+	−	+	−	+	ALT
	shATRX-3	−	+	28	+	−	+	−	+	ALT
	shATRX-4	−	+	21	+	−	+	−	+	ALT
	shDAXX	+	−	0	+	−	+	−	−	ALT

a Protein: ATRX and DAXX protein expression was determined by Western blot.

b Days in crisis: “*” indicates culture did not become immortalized.

c C-circles: “+” indicates the presence of C-circles.

d TRAP: “+” indicates detectable telomerase activity measured by the telomeric repeat amplification protocol (TRAP) assay.

e TRF: “+” indicates telomere length characteristic of ALT-positive cells as determined by the Southern blot-based terminal restriction fragment (TRF) assay.

f TLM: telomere lengthening mechanism as determined by C-circle, TRAP and/or TRF results; ALT and TEL indicate ALT-positive and telomerase-positive, respectively; “-” indicates the culture did not become immortal.

Abbreviation: nd, not determined.

### ATRX knockdown in fibroblasts increases the proportion of cells activating ALT

**Figure 3:**
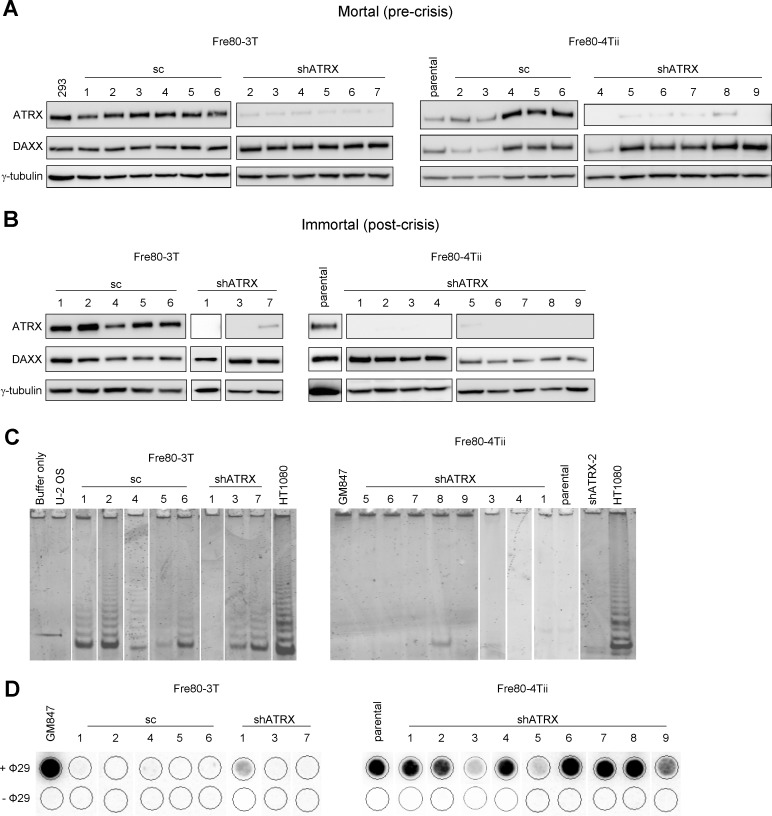
ATRX loss promotes ALT activation in breast fibroblasts **A.** ATRX and DAXX protein expression was analyzed in mortal (pre-crisis) Fre80-3T and Fre80-4Tii fibroblasts transduced with the indicated vectors, as well as untransduced parental Fre80-4Tii cells. 293 cells were included as a positive control and γ-tubulin was used as a loading control. **B.** all immortal cultures were assessed for the levels of ATRX and DAXX protein expression by Western blot. γ-tubulin was used as a loading control and HT1080 cells were used as a positive control. **C.** all immortal fibroblasts lines were examined for the presence of telomerase activity using the TRAP assay. GM847 and U-2 OS cells were used as negative controls and HT1080 cells used as a positive control. **D.** the presence of C-circles was determined in each immortal cell line. GM847 cells were included as a positive control. Samples with C-circle levels above background (−Φ29 negative control) were regarded as ALT-positive.

Since we did not obtain any immortal epithelial cell lines that had activated the ALT mechanism, we next examined the effect of ATRX depletion in fibroblasts. The prevalence of ALT in tumors is skewed towards those of mesenchymal origin [23-25]. We therefore determined whether fibroblasts derived from the same breast sample as Bre80 epithelial cells, with a similar genetic background, had a greater propensity toward ALT activation when ATRX expression was depleted. Two independent SV40-transformed, mortal breast fibroblast cultures, Fre80-3T and Fre80-4Tii, were transduced with either a vector encoding a scrambled shRNA sequence or an shATRX vector. Transduced cells and untransduced (parental) controls were maintained in culture until cultures either escaped from crisis and became immortal, or failed to escape from crisis and became non-viable. ATRX and DAXX proteins were both expressed by each control (scrambled shRNA or parental) culture, and ATRX was efficiently suppressed in each shATRX culture at a pre-crisis time point (Figure 3A). Six out of 13 control cell strains became immortalized, whereas 12 of 16 shATRX-transduced cultures became immortal (Table 1). Expression of ATRX and DAXX proteins in the immortalized cultures (Figure 3B) remained similar to that of pre-crisis cells. All immortal cultures exhibited a period of crisis, and the mean length of crisis between control and shATRX cultures was not significantly different (50 vs 52 days; Table 1). The TLM of each immortal culture was determined using the TRAP and C-circle assays. Of six control cultures that became immortal, five cultures exhibited telomerase activity by the TRAP assay, while the remaining culture was negative for telomerase activity (Figure 3C). Only two of 12 immortal shATRX cell lines showed telomerase activity. The results of the C-circle assay correlated inversely with those of the TRAP assay: each TRAP positive sample was negative for C-circles, and *vice versa* (Figure 3D). The results demonstrate that the induced loss of ATRX significantly promotes ALT activation, as 10 of 12 shATRX-transduced cultures activated the ALT mechanism, while only one of six control cultures was ALT-positive (*P* = 0.01, Fisher's exact test). These data provide the first functional evidence that, in fibroblasts, ATRX loss facilitates ALT activation.

### ATRX knockdown decreases the time required for occurrence of immortalization

**Figure 4:**
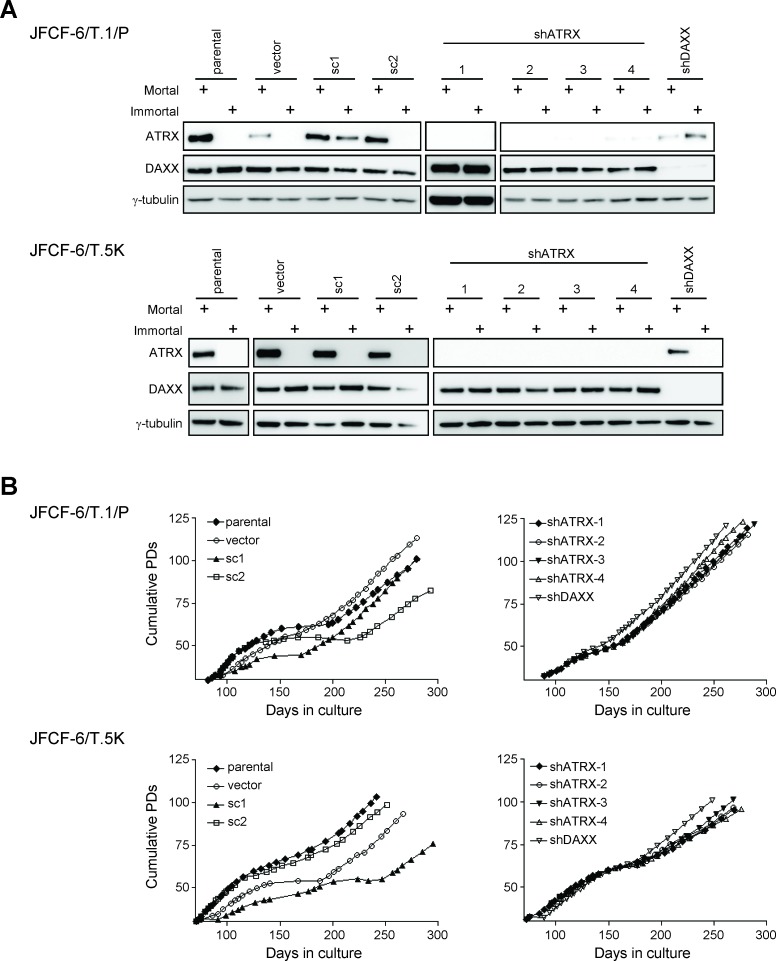
Spontaneous loss of ATRX during immortalization **A.** JFCF-6/T.1/P and JFCF-6/T.5K cells that were unmodified (parental) or transduced with an empty vector (vector), a scrambled shRNA control (sc), shATRX or shDAXX were analyzed by Western blot for the expression of ATRX and DAXX proteins. All cultures were analyzed at a mortal and immortal time point, as indicated by the “+” above each lane. γ-tubulin was used as a loading control. **B.** growth curves of each immortalized JFCF-6/T.1/P and JFCF-6/T.5K cell line. The days in culture and cumulative PDs were calculated subsequent to SV40 transformation.

We then depleted ATRX in two clonal SV40-transformed pre-crisis fibroblast strains from a different source. In addition, we also knocked down DAXX, as both proteins act together as chromatin remodelers and one or both is mutated in pancreatic neuroendocrine tumors with an ALT-like phenotype [6, 26]. ATRX and DAXX proteins were expressed by both pre-crisis strains (JFCF-6/T.1/P and JFCF-6/T.5K) (Figure 4A, lanes labeled parental and mortal). shATRX and shDAXX lentivirus were used to efficiently knock down ATRX or DAXX in both fibroblast cell strains (Figure 4A, shATRX and shDAXX mortal samples). Transduction with the empty vector (vector) or scrambled shRNA control (sc) did not affect endogenous ATRX or DAXX expression. Each mortal culture was passaged through a period of crisis until it became immortal. Growth curves were plotted for each cell line to examine whether there was a change in the length of crisis in shATRX or shDAXX cultures compared to controls (Figure 4B). Six out of eight control cultures showed a distinct period of crisis, ranging from 13 to 78 days (Table 1). Compared to immortal control cultures, shATRX- or shDAXX-transduced cell lines became immortalized after a significantly reduced length of time in crisis (range: 0 to 28 days; *P* < 0.05, Mann Whitney test).

### Spontaneous loss of ATRX expression is also associated with the activation of ALT

ATRX and DAXX protein expression was analyzed in each immortal JFCF-6 cell line (Figure 4A, immortal lanes). ATRX expression was spontaneously lost in 7 of 8 immortal control cultures, as well as in one immortal shDAXX culture. In contrast, spontaneous loss of DAXX was not observed in any immortal culture. ATRX knockdown was maintained in all shATRX-transduced cultures after they became immortalized. Similarly, substantial knockdown of DAXX was maintained after immortalization of both shDAXX-transduced cultures. We sequenced all 35 exons of ATRX to determine whether ATRX protein loss was due to mutation, and identified a premature stop codon in two cell lines that spontaneously lost ATRX expression (ATRX exon 9 of the JFCF-6/T.5K-vector cell line and ATRX exon 10 of the JFCF-6/T.5K-shDAXX culture). The ATRX sequence was wild-type in the remaining six immortal cultures that spontaneously lost ATRX expression, indicating that in these cells ATRX protein is not expressed for reasons other than changes in the coding sequence.

**Figure 5:**
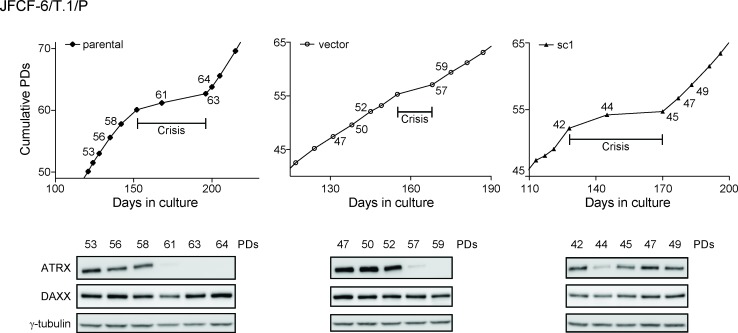
ATRX loss corresponds to a period of growth crisis ATRX and DAXX protein levels of three immortal JFCF-6/T.1/P cell lines (parental, vector and sc1) were analyzed at multiple PDs indicated on the corresponding growth curves; crisis was defined as the cell culture undergoing less than 1 PD/7 days. γ-tubulin was used as a loading control.

We examined the temporal correlation between spontaneous loss of ATRX expression and crisis in three JFCF-6/T.1/P lines, two of which (unmodified parental and vector-transduced) spontaneously lost, and one of which (sc1) maintained ATRX protein expression after immortalization (Figure 5). In both JFCF-6/T.1/P-parental and -vector lines, spontaneous loss of ATRX occurred early during culture crisis. In contrast, the JFCF-6/T.1/P-sc1 culture maintained ATRX expression through crisis. These data demonstrate that spontaneous loss of ATRX can be an early event in the process of cellular immortalization.

**Figure 6:**
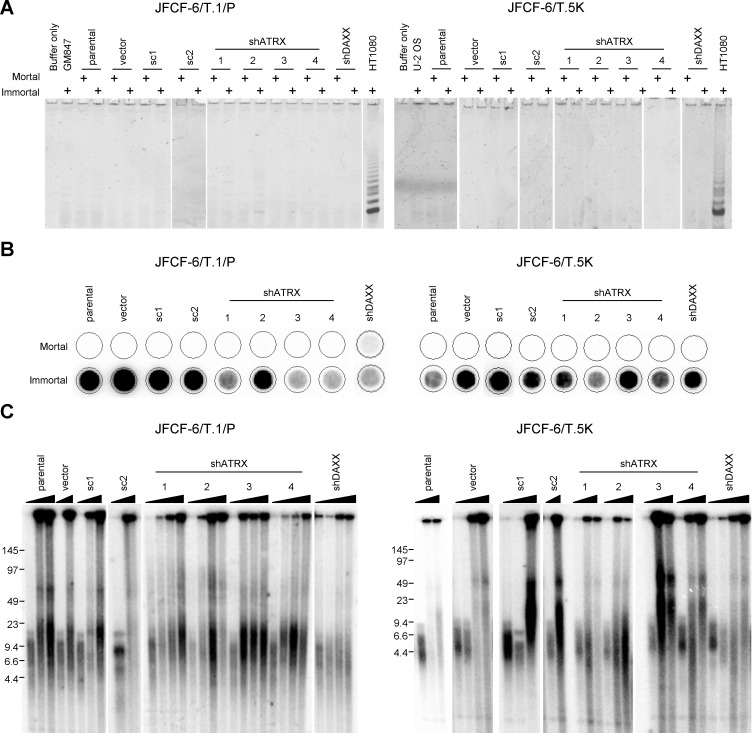
Loss of ATRX promotes fibroblast immortalization TLM status of JFCF-6/T.1/P and JFCF-6/T.5K cell lines treated as described for Figure 4 was analyzed. **A.** telomerase activity was assessed using the TRAP assay. Each sample was assessed at both mortal and immortal time points, indicated by the “+” above each lane. GM847 or U-2 OS cell lysates were used as negative controls and HT1080 served as a positive control, **B.** the presence of C-circles was assessed at both mortal and immortal time points in each cell line. **C.** mean telomere length analysis using the TRF assay was performed on each cell line for at least two time points (mortal and immortal). The triangle above each set of samples indicates increasing PDs.

The TLM that was activated in each immortal JFCF-6/T.1/P- and JFCF-6/T.5K-derived culture was assessed. Every culture was negative for telomerase activity, both before and after immortalization, as demonstrated by the TRAP assay (Figure 6A). All cultures were negative for C-circles prior to immortalization and all post-crisis cultures were C-circle positive (Figure 6B). Telomere length was assessed by Southern blot in each cell line at a minimum of two time points, before and after immortalization, and in each case the immortalized cultures exhibited the heterogeneous telomere length pattern characteristic of ALT, contrasting with the more homogeneous telomere lengths in the pre-crisis cells (Figure 6C). Thus, all immortal JFCF-6-derived fibroblast cell lines activated the ALT mechanism: eight control and 10 shATRX/shDAXX cultures. Spontaneous loss of ATRX was observed in 7 of 8 control cultures and in one shDAXX cell line. These data are clear confirmation that ATRX loss facilitates ALT activation in fibroblasts.

### ATRX expression represses the ALT phenotype

**Figure 7:**
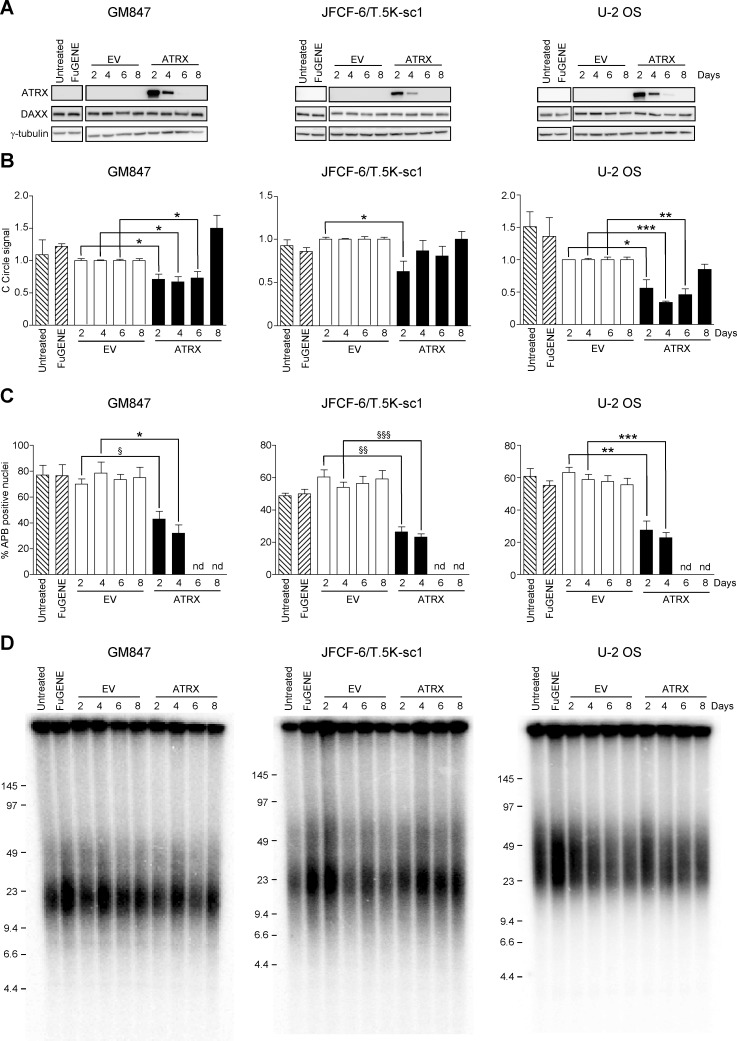
ATRX expression represses the ALT mechanism **A**. ATRX and DAXX protein analysis of untreated control, FuGENE-treated, empty vector (EV)- or ATRX-transfected cultures at days 2, 4, 6 and 8 post-transfection in GM847, JFCF-6/T.5K-sc1 or U-2 OS cells. γ-tubulin was used as a loading control. The blots shown are representative of at least three separate transfections. **B**. the level of C-circles was assessed in each untreated control, FuGENE-treated, EV- or ATRX-transfected GM847, JFCF-6/T.5K-sc1 or U-2 OS cell culture at 2, 4, 6 and 8 days post-transfection. C-circle levels were normalized to the quantity of DNA used for each reaction, followed by normalization to the EV-transfected control at the relevant day post-transfection. Bars indicate the mean ± SEM; n = 3. *P < 0.05, **P < 0.005 and ***P < 0.0001. **C**. APB-positive nuclei were quantified in untreated, FuGENE-treated and EV- or ATRX-transfected GM847, JFCF-6/T.5K-sc1 or U-2 OS cells at the indicated day post-transfection. A nucleus was scored APB-positive when TRF2 and PML co-localized; ATRX/APB-positive nuclei also showed nuclear ATRX staining. The bars represent the mean ± SEM; *n* = 3 to 5; at least 100 nuclei were counted in the untreated, FuGENE-treated or EV-transfected cultures and at least 100 ATRX-positive nuclei were counted in the ATRX-transfected cultures. *P < 0.05, §P < 0.01, **P < 0.005, ^§§^P < 0.001, ***P < 0.0005, ^§§§^P < 0.0001. nd = not determined due to lack of ATRX-positive nuclei. **D**. mean telomere length analysis using the TRF assay in GM847, JFCF-6/T.5K-sc1 or U-2 OS cell cultures treated as indicated above each lane.

Given that the loss of ATRX promoted activation of the ALT mechanism in fibroblasts, we wanted to determine whether restoration of ATRX expression would repress the ALT phenotype. Exogenous ATRX was expressed in three ALT cell lines of mesenchymal origin that lack ATRX expression (GM847, JFCF-6/T.5K-sc1 and U-2 OS). ATRX protein expression was examined at 2, 4, 6 and 8 days following transfection with an empty vector (EV) or an ATRX expression construct. Maximal ATRX protein expression was observed 2 days following transfection, and decreased rapidly, returning to undetectable levels by day 6 in all cell lines examined (Figure 7A). Exogenous expression of ATRX did not affect DAXX expression in any cell line. Furthermore, ATRX transfection did not affect the growth rate or cell cycle kinetics in any of the three cell lines tested (data not shown).

As C-circle levels are indicative of ALT and rapidly respond to perturbations in ALT activity [22], we determined whether C-circle levels changed in response to ATRX transfection. The level of C-circles was significantly reduced following ATRX expression in each of the three cell lines examined compared to untreated, FuGENE-treated or EV-transfected cells (Figure 7B).

APBs are highly correlative with the ALT mechanism [27], and previous studies have demonstrated inhibition of ALT activity results in a decrease in APBs [28]. Therefore, as a further indication that ATRX expression affects the ALT phenotype, APBs were quantified in ALT cells transiently expressing ATRX. The percentage of ATRX-positive nuclei that also contained APBs was significantly reduced compared to the level of APB-positive nuclei in EV-transfected cultures at days 2 and 4 in all cell lines examined (Figure 7C). By day 6, there were insufficient ATRX-positive nuclei to count ATRX-positive/APB-positive nuclei. These results provide further direct evidence that ATRX represses the ALT mechanism.

As an additional control, GM847 cells were transfected either with an EV or a plasmid encoding GFP. The percentage of GM847 nuclei that were positive for both GFP and APBs (80, 81 and 80% at days 2, 4 and 6, respectively) was not significantly different to the percentage of APB-positive nuclei in an EV-transfected culture (75, 87 and 81% at days 2, 4 and 6, respectively). These data confirm that the percentage of nuclei containing APBs was not affected by expression of an irrelevant exogenous protein.

We also examined whether transient ATRX expression affected mean telomere length or the overall telomere length heterogeneity. Neither the mean telomere length nor the telomere length heterogeneity of GM847, JFCF-6/T.5K-sc1 or U-2 OS cells was affected by the transient expression of ATRX (Figure 7D). Therefore rapid changes in telomere length did not occur in response to transient ATRX expression in ALT cells, and long-term ATRX expression may be required for changes in telomere length to occur.

## DISCUSSION

Mutations in members of the ATRX/DAXX chromatin remodeling complex have been implicated in the ALT mechanism, in both ALT-positive cell lines and tumor samples [6, 7, 11, 23]. Although a previous study was unable to demonstrate immortalization of SV40-transformed BJ fibroblasts following ATRX knockdown [7], we found that depletion of ATRX facilitates immortalization of fibroblasts, increasing the proportion of cells that activate ALT compared to telomerase activation (Fre80 cultures) and decreasing the time in crisis prior to immortalization (JFCF-6 cultures). There was no evidence that the effect of ATRX depletion was different in these two sets of cultures: there was an insufficient number of Fre80 cultures which spontaneously activated ALT in the absence of ATRX knockdown to see a significant effect on length of crisis and, conversely, all of the spontaneously immortalized JFCF-6 cultures in this study activated ALT, making it impossible to observe an increased proportion of ALT-positive cultures following ATRX knockdown. The data are consistent with the hypotheses that 1) activation of ALT and telomerase in the context of inactivated p53 and pRb/p16INK4a tumor suppressor pathways each requires at least two genetic or epigenetic events, 2) the events are different for the two TLMs, and 3) one of the events for activating ALT is the loss of ATRX function. In pre-crisis cells that have undergone none of the events required for activation of either ALT or telomerase, experimentally depleting ATRX would result in fewer additional events being required for activation of ALT, which would both increase the probability that ALT becomes activated and decrease the time required for immortalization to occur.

Spontaneous ATRX loss was observed in eight immortal JFCF-6 fibroblast cultures; the time course of ATRX loss was examined in two of these cultures, and in both cases this occurred early in crisis. This is also consistent with the hypothesis that ATRX loss-of-function is one of at least two genetic events required for escape from crisis via activation of ALT. It is not possible to conclude from this limited number of observations whether ATRX loss is usually the first event, possibly contributing to the probability that the additional event(s) will occur, or whether the order of the events is stochastic. The reason for spontaneous ATRX loss was determined to be a mutation that resulted in a premature stop codon in two immortal lines. The absence of an exonic mutation in the other lines is consistent with a previous report of 12 ALT-positive cell lines (including two JFCF-6 lines) that had wild-type ATRX sequence but lacked or had abnormal ATRX protein expression [7]. Absence of ATRX protein expression in the presence of wild-type ATRX sequence may potentially be due to epigenetic changes, miRNA targeting or post-translational modifications. The presence of ATRX expression in a small number of ALT-positive cell lines suggests that there are other proteins whose loss can have the same functional outcome as loss of ATRX expression. The higher frequency of ALT activation in JFCF-6 compared to Fre80 cells may indicate that the respective pre-crisis cultures may have accumulated different pre-disposing genetic or epigenetic events. Nevertheless, the activation of ALT in all cell lines that spontaneously lost ATRX provides additional evidence for its role as an ALT suppressor.

We also showed that successful knockout of ATRX using CRISPR/Cas9 technology or rAAV in epithelial-derived HCT116 colon carcinoma cells did not affect telomerase expression nor activate ALT. This indicates that telomerase activity does not require the presence of functional ATRX. The lack of any ALT immortalized epithelial cell lines in the breast epithelial cell experiments, in contrast to the results with breast stromal fibroblasts derived from the same individual, is consistent with the observation that ALT-positive tumors are more frequently of mesenchymal, rather than epithelial, origin [29] and support the concept that there are inherent cell type differences, and possibly requirements, with regard to cellular immortalization [30]. The genetic events required to activate telomerase in cell types, including epithelia, where low levels of telomerase activity occur physiologically, may differ from the events required to activate telomerase in cell types such as fibroblasts where telomerase activity is normally undetectable, and presumably tightly repressed. If this is the case, then in epithelial cells where ATRX is experimentally depleted, the probability of the additional events required to activate telomerase occurring may still be greater than the probability of activating ALT.

Transient ATRX expression repressed the ALT mechanism in three separate ALT-positive/ATRX-negative cell lines. This is the first functional evidence that ATRX expression is able to repress the ALT mechanism. ALT repression clearly resulted from ATRX expression, as when ATRX was expressed, both C-circle levels and the proportion of APB positive nuclei decreased. Both C-circle and APB levels returned to control levels following the loss of ATRX protein overexpression. The mechanism by which ATRX represses the phenotypic ALT markers is unknown but may include facilitating replication and resolving G-quadruplex secondary structures [15]. An attractive alternative hypothesis is that the chromatin remodeling capabilities of ATRX are critical, as alterations in the heterochromatic state of telomeres and chromatin-associated proteins have been implicated in the ALT mechanism [31-34]. For instance, silencing of the histone chaperone ASF1 induces the ALT phenotype [33] and the NuRD-ZNF827 complex is present at ALT telomeres and causes histone hypoacetylation, a marker of closed chromatin [31]. Confusingly however, decondensation of telomeric chromatin, as determined by micrococcal nuclease digestion, has also been shown in ALT cells [32]. Despite these somewhat contradictory results implying that ALT telomeres may be more heterochromatic or euchromatic depending upon the model system utilized, a consistent feature is that chromatin is somehow altered in all these cells and that this alteration seems likely causative, rather than simply an indirect effect. Further studies that determine the requirement for altered telomeric chromatin status in ALT, and how chromatin remodelers play an essential role in this process, are likely to yield important insights into the ALT mechanism.

## MATERIALS AND METHODS

### Cell culture

JFCF-6/T.1/P and JFCF-6/T.5K cell strains were derived from independent SV40-transformation events of mortal jejunal fibroblasts from a male cystic fibrosis patient. GM847DM cells (referred to as GM847) were derived from Lesch-Nyhan syndrome human fibroblasts immortalized with SV40 [35]. HT1080, 293 and U-2 OS cells were obtained from the American Type Culture Collection (ATCC). Two separate SV40-transformed breast stromal fibroblast cell strains, Fre80-3T and Fre80-4Tii, were also used in the current studies [36]. All of these strains and lines were cultured in DMEM with 10% fetal bovine serum (FBS). Two individual SV40-transformed Bre80 cell strains, breast stromal epithelial cells derived from the same individual as the Fre80 fibroblasts, were cultured in MCDB-170 serum-free medium (Life Technologies). The HCT116 colon carcinoma cell line (ATCC) and its derivatives were cultured in McCoy's 5A medium supplemented with 10% FBS, penicillin and streptomycin (each 100 U/mL), and 5 mM L-glutamine. All cultures were grown in a humidified incubator at 37°C with 5% CO_2_. Crisis was defined as a time period during which the cell culture underwent less than 1 PD per 7 days. The cell lines were confirmed to be free of Mycoplasma species and the identity confirmed using short tandem repeat profiling by CellBank Australia (Sydney, Australia).

### Gene transfer

#### Lentiviral infection

Lentiviral constructs encoding a scrambled control shRNA in one of two vectors (pLKO.1 or pLKO.5), GFP (pLKO.1 vector), shATRX [constructs 13590 (pLKO.1 vector) and 342811 (pLKO.5 vector)] or shDAXX [construct 3800 (pLKO.1 vector)], as well as the pLKO.1 empty vector were obtained from Sigma. Lentivirus was produced and target cells were infected with equivalent multiplicities of infection. Infected cells were continuously cultured in medium supplemented with 0.5 μg/mL puromycin. Three sublines were established from each JFCF-6/T.1/P- and JFCF-6/T.5K-shATRX-1 mass culture prior to immortalization. The following cell strains/lines were transduced with pLKO.1-scrambled: JFCF-6/T.1/P-sc1, JFCF-6/T.5K-sc1, Bre80 T5 sc, Fre80-3T sc1-3 and Fre80-4Tii sc1-3; the remaining scrambled-shRNA control lines were transduced with a pLKO.5-scrambled shRNA vector. Fre80-3T shATRX-1, Fre80-4Tii shATRX-4, Bre80 T5 shATRX-2 and Bre80 T8 shATRX-3, -4, -5 lines were transduced with shATRX construct 342811, while the remaining shATRX lines were transduced with shATRX construct 13590.

#### Transfection

An ATRX expression vector, pCMV6-Entry-ATRX, the empty vector pCMV6-Entry and a GFP-expressing vector, pCMV6-AC-GFP, were obtained from OriGene. Transfection with either vector was performed using FuGENE reagent (Promega). Cells were harvested at the indicated time points post-transfection and analyzed as described. The mean percentage (± SEM) of ATRX-positive nuclei at day 2 in GM847, JFCF-6/T.5K-sc1 and U-2 OS cells was 50 ± 11, 18 ± 2 and 26 ± 2%, respectively. At day 4, the mean ATRX-positive nuclei percentage (± SEM) in GM847, JFCF-6/T.5K-sc1 and U-2 OS cells was 39 ± 17, 10 ± 2 and 19 ± 4%, respectively.

#### CRISPR/Cas9 ATRX knockout

The ATRX cDNA was screened by ZiFiT to determine a CRISPR target in exon 9 of ATRX [37]. Two complementary oligos were designed by ZiFit, ATRXex9_1 and ATRXex9_2 (Supplementary Table 1), and were subsequently annealed and ligated into a CRISPR RNA expression plasmid (MLM3636; Addgene). The resulting plasmid was co-transfected with a Cas9 nuclease expression plasmid (41815; Addgene) into wild-type HCT116. Cells were subcloned, and screened for correct targeting by interrogating the disruption of an *Sml*I restriction enzyme site that lies directly adjacent to the target sequence cut by the Cas9 endonuclease. Targeting PCR was performed using ATRXex9F and ATRXex9R (Supplementary Table 1), and products were subjected to *Sml*I digestion. Sanger sequencing of the correctly modified clones was performed with ATRXex9SeqF to confirm that an early stop codon was inserted or a frame-shift mutation had occurred.

#### rAAV-mediated ATRX knockout

A knockout rAAV vector was constructed as described, with slight modifications [38-40]. The fifth exon of the ATRX gene was targeted, as it has been reported that disruption of this exon results in an ATRX knockout [41]. Accordingly, the left and right homology arms were constructed by PCR with primer pairs, ATRX LarmF and ATRX LarmR, and with ATRX RarmF and ATRX RarmR, respectively (Supplementary Table 1). These PCR products were combined with a neomycin resistance gene, and an rAAV vector backbone, and used in a Golden Gate ligation reaction to generate the rAAV ATRXKO. Following virus production and infection of wild-type HCT116 cells, neomycin resistant sub-clones were screened by PCR with ATRX TargF and ATRX IresR primers (Supplementary Table 1) to identify correctly targeted clones. Clones were continuously cultured in media containing 1 mg/mL G418.

### Immunoblot analysis

Cell lysate preparation and protein detection was performed as described [11]. Briefly, protein lysates were prepared from harvested cells, separated by electrophoresis using a Tris-acetate gel, transferred to PVDF membrane, and the membrane was probed for protein using the antibodies indicated in each figure. Antibodies used: ATRX (Sigma, HPA001906 or GeneTex, GTX629703), DAXX (Sigma, HPA008736), γ-tubulin (Sigma, T5192) and Ku70 (Santa Cruz, sc-9033).

### APB detection

APB analysis was performed by cytocentrifuging cells onto SuperFrost Plus slides using a Shandon Cytospin 4. Cells were then fixed with 4% formaldehyde and permeabilized with 0.1% Triton X-100. Blocking and RNase A treatment were simultaneously performed by incubating slides with 0.1 mg/mL RNase A diluted in antibody dilution buffer (ABDIL; 20 mM Tris-HCl, pH 7.5, 0.2% fish gelatin, 2% BSA, 0.1% Triton X-100, 150 mM NaCl, 0.1% sodium azide). Cells were stained with antibodies specific for ATRX (Sigma, HPA001906), TRF2 (Merck Millipore, OP129) and PML (Santa Cruz, sc-9862) diluted in ABDIL for 2 h at 37ÐC in a humidified chamber. Following washes in PBS with 0.1% Tween-20 (PBST), cells were stained with appropriate AlexaFluor secondary antibodies for 30 min at 37ÐC in a humidified chamber. Subsequent to an additional set of PBST washes, nuclei were counterstained with DAPI and mounted using DABCO anti-fade mounting media. Images were obtained on an Imager. M1 microscope (Zeiss) and analysis performed using ZEN software (Zeiss). Statistical analyses were performed using GraphPad Prism 5.0.

### C-circle assay

The C-circle assay was performed as described [22]. C-circle signal was quantified using ImageQuant software and background corrected by edge subtraction. Quantitation of the C-circle signal in the ATRX expression experiments was performed by normalizing the signal obtained for each sample to the amount of DNA used per reaction, followed by comparison to the signal obtained on the corresponding day post-transfection with the empty vector pCMV6-Entry.

### Terminal restriction fragment (TRF) assay

Telomere length was assessed essentially as described [11]. Equivalent amounts of genomic DNA were digested with *Hinf*I and *Rsa*I and separated using either standard or pulsed field gel electrophoresis. The resulting gel was hybridized either in-gel or after transfer to Hybond XL (GE Healthcare) membrane using a radioactive (TTAGGG)_3_ oligonucleotide probe. Detection of radioactive signal was performed using a phosphor-imaging screen and the signal measured using a Typhoon Trio (GE Healthcare).

### Telomere repeat amplification protocol (TRAP)

The TRAP assay was used to assess telomerase activity as described [11]. Lysates were prepared using CHAPS buffer and equal amounts of lysate were used in each TRAP reaction. The TRAP products were separated on a PAGE gel and the gel was stained with SYBR Gold. Gels were scanned on a Typhoon Trio.

### ATRX sequencing

ATRX was sequenced using described primers and conditions [42]. PCR products were purified and sent to the Australian Genome Research Facility for Sanger sequencing. Sequence analysis was performed using CodonCode Aligner.

## SUPPLEMENTAl MATERIAL TABLE



## References

[1] Harley CB, Futcher AB, Greider CW (1990). Telomeres shorten during ageing of human fibroblasts. Nature.

[2] Greider CW, Blackburn EH (1985). Identification of a specific telomere terminal transferase activity in Tetrahymena extracts. Cell.

[3] Bryan TM, Englezou A, Gupta J, Bacchetti S, Reddel RR (1995). Telomere elongation in immortal human cells without detectable telomerase activity. EMBO J.

[4] Reddel RR (2014). Telomere maintenance mechanisms in cancer: clinical implications. Curr Pharm Des.

[5] Perrem K, Bryan TM, Englezou A, Hackl T, Moy EL, Reddel RR (1999). Repression of an alternative mechanism for lengthening of telomeres in somatic cell hybrids. Oncogene.

[6] Heaphy CM, de Wilde RF, Jiao Y, Klein AP, Edil BH, Shi C, Bettegowda C, Rodriguez FJ, Eberhart CG, Hebbar S, Offerhaus GJ, McLendon R, Rasheed BA (2011). Altered telomeres in tumors with ATRX and DAXX mutations. Science.

[7] Lovejoy CA, Li W, Reisenweber S, Thongthip S, Bruno J, de Lange T, De S, Petrini JH, Sung PA, Jasin M, Rosenbluh J, Zwang Y, Weir BA (2012). Loss of ATRX, genome instability, and an altered DNA damage response are hallmarks of the Alternative Lengthening of Telomeres pathway. PLoS Genet.

[8] Cheung NK, Zhang J, Lu C, Parker M, Bahrami A, Tickoo SK, Heguy A, Pappo AS, Federico S, Dalton J, Cheung IY, Ding L, Fulton R (2012). Association of age at diagnosis and genetic mutations in patients with neuroblastoma. JAMA.

[9] Chen X, Bahrami A, Pappo A, Easton J, Dalton J, Hedlund E, Ellison D, Shurtleff S, Wu G, Wei L, Parker M, Rusch M, Nagahawatte P (2014). Recurrent somatic structural variations contribute to tumorigenesis in pediatric osteosarcoma. Cell Rep.

[10] Schwartzentruber J, Korshunov A, Liu XY, Jones DT, Pfaff E, Jacob K, Sturm D, Fontebasso AM, Quang DA, Tonjes M, Hovestadt V, Albrecht S, Kool M (2012). Driver mutations in histone H3. 3 and chromatin remodelling genes in paediatric glioblastoma. Nature.

[11] Bower K, Napier CE, Cole SL, Dagg RA, Lau LM, Duncan EL, Moy EL, Reddel RR (2012). Loss of wild-type ATRX expression in somatic cell hybrids segregates with activation of Alternative Lengthening of Telomeres. PLoS ONE.

[12] Xue Y, Gibbons R, Yan Z, Yang D, McDowell TL, Sechi S, Qin J, Zhou S, Higgs D, Wang W (2003). The ATRX syndrome protein forms a chromatin-remodeling complex with Daxx and localizes in promyelocytic leukemia nuclear bodies. Proc Natl Acad Sci U S A.

[13] Lukashchuk V, Everett RD (2010). Regulation of ICP0-null mutant herpes simplex virus type 1 infection by ND10 components ATRX and hDaxx. J Virol.

[14] Ritchie K, Seah C, Moulin J, Isaac C, Dick F, Berube NG (2008). Loss of ATRX leads to chromosome cohesion and congression defects. J Cell Biol.

[15] Law MJ, Lower KM, Voon HP, Hughes JR, Garrick D, Viprakasit V, Mitson M, De Gobbi M, Marra M, Morris A, Abbott A, Wilder SP, Taylor S (2010). ATR-X syndrome protein targets tandem repeats and influences allele-specific expression in a size-dependent manner. Cell.

[16] Girardi AJ, Jensen FC, Koprowski H (1965). SV40-induced transformation of human diploid cells: crisis and recovery. J Cell Comp Physiol.

[17] Counter CM, Avilion AA, LeFeuvre CE, Stewart NG, Greider CW, Harley CB, Bacchetti S (1992). Telomere shortening associated with chromosome instability is arrested in immortal cells which express telomerase activity. EMBO J.

[18] Ide T, Tsuji Y, Nakashima T, Ishibashi S (1984). Progress of aging in human diploid cells transformed with a tsA mutant of simian virus 40. Exp Cell Res.

[19] Huschtscha LI, Holliday R (1983). Limited and unlimited growth of SV40-transformed cells from human diploid MRC-5 fibroblasts. J Cell Sci.

[20] Colgin LM, Reddel RR (1999). Telomere maintenance mechanisms and cellular immortalization. Curr Opin Genet Dev.

[21] Chen B, Gilbert LA, Cimini BA, Schnitzbauer J, Zhang W, Li GW, Park J, Blackburn EH, Weissman JS, Qi LS, Huang B (2013). Dynamic imaging of genomic loci in living human cells by an optimized CRISPR/Cas system. Cell.

[22] Henson JD, Cao Y, Huschtscha LI, Chang AC, Au AY, Pickett HA, Reddel RR (2009). DNA C-circles are specific and quantifiable markers of alternative-lengthening-of-telomeres activity. Nat Biotechnol.

[23] Henson JD, Reddel RR (2010). Assaying and investigating Alternative Lengthening of Telomeres activity in human cells and cancers. FEBS Lett.

[24] Heaphy CM, Subhawong AP, Hong SM, Goggins MG, Montgomery EA, Gabrielson E, Netto GJ, Epstein JI, Lotan TL, Westra WH, Shih IM, Iacobuzio-Donahue CA, Maitra A (2011). Prevalence of the alternative lengthening of telomeres telomere maintenance mechanism in human cancer subtypes. Am J Pathol.

[25] Ulaner GA, Huang HY, Otero J, Zhao Z, Ben-Porat L, Satagopan JM, Gorlick R, Meyers P, Healey JH, Huvos AG, Hoffman AR, Ladanyi M (2003). Absence of a telomere maintenance mechanism as a favorable prognostic factor in patients with osteosarcoma. Cancer Res.

[26] Lewis PW, Elsaesser SJ, Noh KM, Stadler SC, Allis CD (2010). Daxx is an H3. 3-specific histone chaperone and cooperates with ATRX in replication-independent chromatin assembly at telomeres. Proc Natl Acad Sci U S A.

[27] Yeager TR, Neumann AA, Englezou A, Huschtscha LI, Noble JR, Reddel RR (1999). Telomerase-negative immortalized human cells contain a novel type of promyelocytic leukemia (PML) body. Cancer Res.

[28] Jiang WQ, Zhong ZH, Henson JD, Neumann AA, Chang AC, Reddel RR (2005). Suppression of alternative lengthening of telomeres by Sp100-mediated sequestration of MRE11/RAD50/NBS1 complex. Mol Cell Biol.

[29] Henson JD, Neumann AA, Yeager TR, Reddel RR (2002). Alternative lengthening of telomeres in mammalian cells. Oncogene.

[30] Lafferty-Whyte K, Cairney CJ, Will MB, Serakinci N, Daidone MG, Zaffaroni N, Bilsland A, Keith WN (2009). A gene expression signature classifying telomerase and ALT immortalization reveals an hTERT regulatory network and suggests a mesenchymal stem cell origin for ALT. Oncogene.

[31] Conomos D, Reddel RR, Pickett HA (2014). NuRD-ZNF827 recruitment to telomeres creates a molecular scaffold for homologous recombination. Nat Struct Mol Biol.

[32] Episkopou H, Draskovic I, van Beneden A, Tilman G, Mattiussi M, Gobin M, Arnoult N, Londono-Vallejo A, Decottignies A (2014). Alternative Lengthening of Telomeres is characterized by reduced compaction of telomeric chromatin. Nucleic Acids Res.

[33] O'Sullivan RJ, Arnoult N, Lackner DH, Oganesian L, Haggblom C, Corpet A, Almouzni G, Karlseder J (2014). Rapid induction of alternative lengthening of telomeres by depletion of the histone chaperone ASF1. Nat Struct Mol Biol.

[34] Jiang WQ, Nguyen A, Cao Y, Chang AC, Reddel RR (2011). HP1-mediated formation of Alternative Lengthening of Telomeres-associated PML bodies requires HIRA but not ASF1a. PLoS ONE.

[35] Pereira-Smith OM, Smith JR (1988). Genetic analysis of indefinite division in human cells: identification of four complementation groups. Proc Natl Acad Sci U S A.

[36] Kaul Z, Cesare AJ, Huschtscha LI, Neumann AA, Reddel RR (2012). Five dysfunctional telomeres predict onset of senescence in human cells. EMBO Rep.

[37] Hwang WY, Fu Y, Reyon D, Maeder ML, Tsai SQ, Sander JD, Peterson RT, Yeh JR, Joung JK (2013). Efficient genome editing in zebrafish using a CRISPR-Cas system. Nat Biotechnol.

[38] Khan IF, Hirata RK, Russell DW (2011). AAV-mediated gene targeting methods for human cells. Nat Protoc.

[39] Kohli M, Rago C, Lengauer C, Kinzler KW, Vogelstein B (2004). Facile methods for generating human somatic cell gene knockouts using recombinant adeno-associated viruses. Nucleic Acids Res.

[40] Luo Y, Lin L, Bolund L, Sorensen CB (2014). Efficient construction of rAAV-based gene targeting vectors by Golden Gate cloning. Biotechniques.

[41] Leung JW, Ghosal G, Wang W, Shen X, Wang J, Li L, Chen J (2013). Alpha thalassemia/mental retardation syndrome X-linked gene product ATRX is required for proper replication restart and cellular resistance to replication stress. J Biol Chem.

[42] Jiao Y, Shi C, Edil BH, de Wilde RF, Klimstra DS, Maitra A, Schulick RD, Tang LH, Wolfgang CL, Choti MA, Velculescu VE, Diaz LA, Vogelstein B (2011). DAXX/ATRX, MEN1, and mTOR pathway genes are frequently altered in pancreatic neuroendocrine tumors. Science.

