# Supplementary material for: ATRX represses alternative lengthening of telomeres
Source: Oncotarget. 2015 Apr 15;6(18):16543–58. doi: 10.18632/oncotarget.3846 (PMC4599288; doi:10.18632/oncotarget.3846)
Supplement: Supplementary file 1 [file oncotarget-06-16543-s001.pdf]

ATRX represses alternative lengthening of telomeres

Supplementary Material

Supplementary Table 1: Oligo sequences used to construct and verify ATRX knockout cell lines.

Oligo name	Sequence (5' – 3')
ATRXex9_1	ACACCGTTTCTGTCGGTCGCCTCAAG
ATRXex9_2	AAAACTTGAGGCGACCGACAGAAACG
ATRXex9F	AGTGGAACTGAACAAGAAGTGG
ATRXex9R	CGTGTCTTTATCAACTGTGCCTTC
ATRXex9SeqF	CCTGTTTCCCTTTCTAATTCCC
ATRX LarmF	ATACATAGCGGCCGCAGGTCCTTTGCCTGATACAC
ATRX LarmR	TATTACTAGTGACCCTGGAGGACACTCTTC
ATRX RarmF	ATAACTCGAGGATGAGAATGACAGGGAACTTGG
ATRX RarmR	ATATTGCGGCCGCGTGAATTGAAGAGAGAGGCTGT
ATRX TargF	GAGGTGGATGTTAAGGGCATG
ATRX IresR	GCTTCCAGAGGAACTGCTTCC
ATRX IntF	CTAACCAGTGGGAATGTCTC
ATRX IntR	CAGGGAACAAGGTTTAATAAGCAC
